# Age and sex distribution of *Dirofilaria immitis* among dogs in Meshkin-Shahr, northwest Iran and molecular analysis of the isolates based on COX1 gene

**Published:** 2016-12-15

**Authors:** Zabiholah Zarei, Eshrat Beigom Kia, Zahra Heidari, Fattaneh Mikaeili, Mehdi Mohebali, Meysam Sharifdini

**Affiliations:** 1 Department of Medical Parasitology and Mycology, School of Public Health, Tehran University of Medical Sciences, Tehran, Iran; 2 Center for Research of Endemic Parasites of Iran, Tehran University of Medical Sciences, Tehran, Iran; 3 Department of Medical Microbiology, School of Medicine, Ardabil University of Medical Sciences, Ardabil, Iran; 4 Department of Medical Parasitology, School of Medicine, Shiraz University of Medical Sciences, Shiraz, Iran; 5 Center for Research of Endemic Parasites of Iran, Tehran University of Medical Sciences, Tehran, Iran; 6 Department of Medical Microbiology, School of Medicine, Guilan University of Medical Sciences, Rasht, Iran

**Keywords:** *Dirofilaria immitis*, Dog, Meshkin-Shahr, Molecular analysis

## Abstract

*Dirofilaria immitis* is an important filarial nematode in dogs. In this study, age and sex distribution of this zoonotic nematode among dogs were investigated in northwest of Iran in Meshkin-Shahr city. Molecular characteristics of the isolates, based on cytochrome oxidase subunit 1 (COX1) gene were compared to the isolates from other areas of the world.Blood samples were collected from 91 dogs which were selected by simple classified accidental sampling. Thin and thick blood smear examinations were used to find out infectivity with *D. immitis*. DNA extraction was performed from adult *D. immitis* recovered from heart of infected dogs. The COX1 gene was amplified and sequenced. Phylogenetic analysis was carried out using sequences obtained in this study along with relevant sequences deposited in the GenBank. Phylogenetic analysis and sequence variation was performed using MEGA software in comparison with those COX1 sequences deposited in GenBank. Out of 91 dogs, 19 (20.87%) were found positive for infection with *D. immitis*. There was no statistically significant difference between males and females of dogs in terms of *D. immitis* infection. However, the rate of infection in dogs more than 2 years old was significantly higher than those with lower age. Both sequences analyzed in this study showed 100% homology to each other. Intra-species variation of these isolates with those from other areas of the world amounted to 0 to 0.50%. Phylogenetic analysis of the COX1 gene suggested that it is conserved, and can be used for study on genetic diversity and classification of filarial nematodes.

## Introduction

*Dirofilaria immitis* is a mosquito born filarial nematode and a wide range of carnivores especially dogs and cats are the known definitive hosts.^1^ It is transmitted by several mosquito species of *Culicidae *family as vector.^2^ Adult worms live in right ventricle, pulmonary artery and posterior vena cava.^3^ Microfilariae circulate in the peripheral vasculatures in dogs while amicrofilaremic infections are common in felines.^1^

Dirofilariosis often has no clinical signs in mild infection, however, may cause clinical signs as cough, congestive heart failure, intravascular haemolysis and pulmonary thromboembolism, and even death in dogs.^1^^,^^4^ In human, *D. immitis* causes pulmonary dirofilariasis and is usually asymptomatic. In symptomatic dirofilariasis cough, chest pain, fever, and pleural effusion are present.^1^^,^^5^

*Dirofilaria immitis* is widespread in the tropics, subtropics and temperate zones.^1^^,^^6^^,^^7^ Canine *D. immitis* infection is reported in different areas of Iran. Epidemiological studies have indicated that the prevalence of *D. immitis* in dogs from different parts of Iran ranges from 1.40% to 51.40%.^8^^-^^17^ Meshkin-Shahr is an endemic area for this parasite. There are four reports of human *D. immitis* infection in Iran in which two pulmonary cases were related to Meshkin-Shahr district.^16^^,^^18^^-^^20^ Regarding high prevalence of dirofilariasis in dogs in this area and their medical importance, the current study was designed to understand age and sex distribution of *D. immitis *in infected dogs and molecular analysis of *D. immitis* based on partial mitochondrial cytochrome oxidase subunit 1 (COX1) sequence and their phylogenetic relationships compared to the isolates from other areas of the world.

## Materials and Methods

**Fig. 1:**
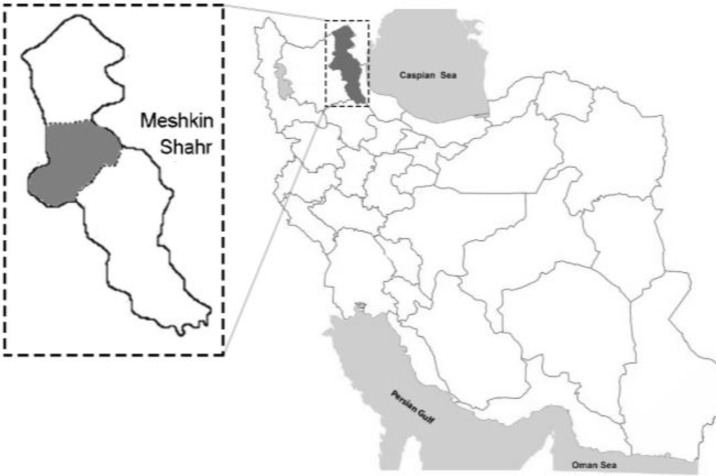
Map of Iran showing geographical location of Ardabil Province and the study area, Meshkin-Shahr

**Study area. **Meshkin-Shahr is a city located in the central northern part of the Ardabil Province, northwest of Iran. It is situated at an altitude of 1490 m above sea level between longitudes 47° 190ˊ and 48° 170ˊ East and latitudes 38° 570ˊ and 38° 130ˊ North (Fig. 1). The relative humidity alters between 61.00% and 70.00% and the annual precipitation varies between 300 and 385 mm. The study area has moderate mountainous weather. In this area, there are numerous domestic dogs in relationship with human population which used as guard and herd dogs.^21^

**Sampling. **Using simple classified accidental sampling, blood samples were taken from the cephalic vein of 91 dogs during spring and summer 2009 to 2011. Sex and age of the dogs were determined by a local veterinary practitioner and recorded.

**Parasitological study. **Thin and thick smears were prepared for each blood sample fixed in absolute methanol and stained with 10% Giemsa. The presence of microfilaria was detected by light microscopy examination of the slides. 

**Molecular and phylogenetic analysis. **Adult worms of *D. immitis* were isolated from two dogs that were simultaneously infected with *Leishmania infantum* and underwent necropsy during a study on visceral leishmaniasis in ownership dogs.^22^ Adult worms were washed extensively in physiological saline after removal from heart and preserved in 70% (v/v) ethanol until extraction of genomic DNA.

At the time of extraction, *D. immitis* adult worms were thoroughly washed in distilled water to remove ethanol. Total genomic DNA was extracted using Bioneer DNA extraction kit (Bioneer Corp., Daejeon, Korea) according to the manufacturer's instructions and stored at – 20 ˚C until polymerase chain reaction (PCR) amplification.

The PCR was carried out in a final reaction volume of 30 μL using 15 μL of PCR mix containing 1.25 U Taq DNA polymerase, 200 μM of dNTPs and 1.5 mM MgCl_2_ (2x Master Mix RED; Ampliqon, Odense, Denmark); 10 pmol of each primer and 3 μL of DNA sample. Primers COIintF (5-TGATTGGTGGTTTTGGTAA-3) and COIintR (5-ATAAGTAC GAGTATCAATATC-3) amplify a 689 bp target of COX1 gene.^23^ The temperature profile was an initial denaturation step at 94 ˚C for 5 min, followed by 30 cycles of denaturation at 94 ˚C for 30 sec, annealing at 52 ˚C for 45 sec, extension at 72 ˚C for 60 sec, followed by a final extension at 72 ˚C for 7 min.

The PCR products were electrophoresed on a 1.5% of agarose gel (Cinnagen Co., Tehran, Iran) and visualized using ethidium bromide in UV transilluminator (Uvitec Co., Cambridge, UK). Next, The PCR products were purified with a commercial purification kit (Bioneer), according to the manufacturer’s instructions. Purified products were sequenced in one direction using the forward primer.

Sequence results were edited and analyzed by Geneious software (Biomatters Ltd., Auckland, New Zealand). The sequences were compared GenBank references sequences by BLAST program.^24^ Phylogenetic analysis was carried out using maximum likelihood method based on Tamura-Nei model.^25^ Pairwise comparisons were determined as as the level of sequence differences using MEGA software (Version 5.0; Biodesign Institute, Tempe, USA).^25^ Bootstrap analysis was carried out with 1000 replications.

**Statistical analysis. **Chi-square (X^2^) test was used to compare infection with *D. immitis* in the infected dogs in association with their gender and age. Statistical analyses were performed using SPSS (version 13.5; SPSS Inc, Chicago, USA), with a *p* value less than 0.05 as statistically significant.

## Results

 Out of 91 dogs examined in the current study, using direct blood smears, 75 (82.40%) were male and 16 (17.60%) were female. All of the dogs were in mixed breed, keeping as guard dogs and sheep-dogs. Overall, 19 dogs (20.87%) were found infected with *D. immitis*. The rate of infection in male and female dogs were 15 (16.40%) and 4 (12.00%), respectively. No statistically significant difference was found between *D. immitis *infection and gender (*p *= 0.70). However, the rate of infection in dogs more than 2 years old (17/60) was significantly higher than those with lower age (2/31) (28.30% versuss 6.40%; *p *= 0.03).

**Fig. 2:**
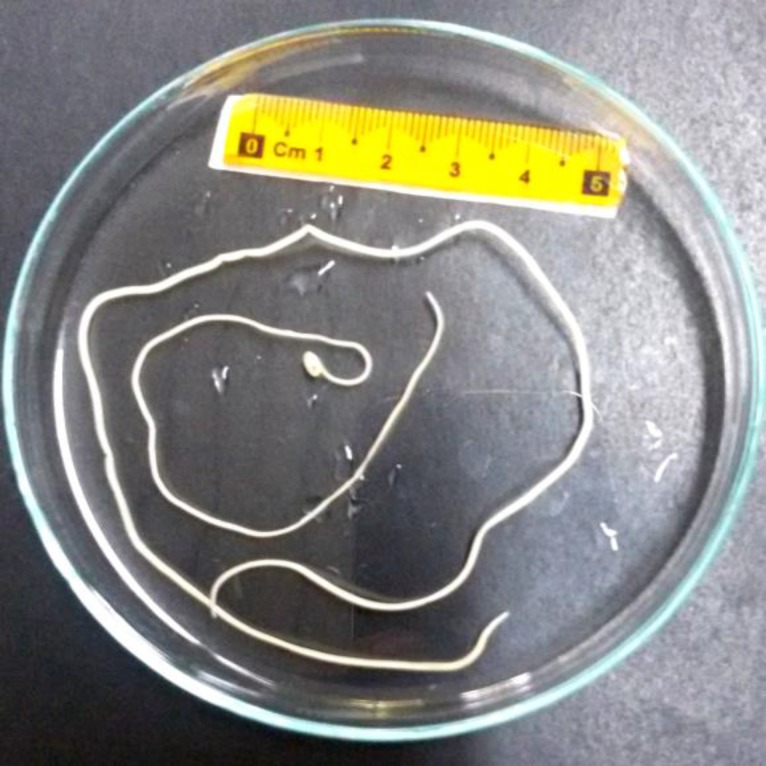
Adult *Dirofilaria immitis*. Male worm is shorter and has a spirally coiled posterior end; the female is larger and straight on both ends

**Fig. 3:**
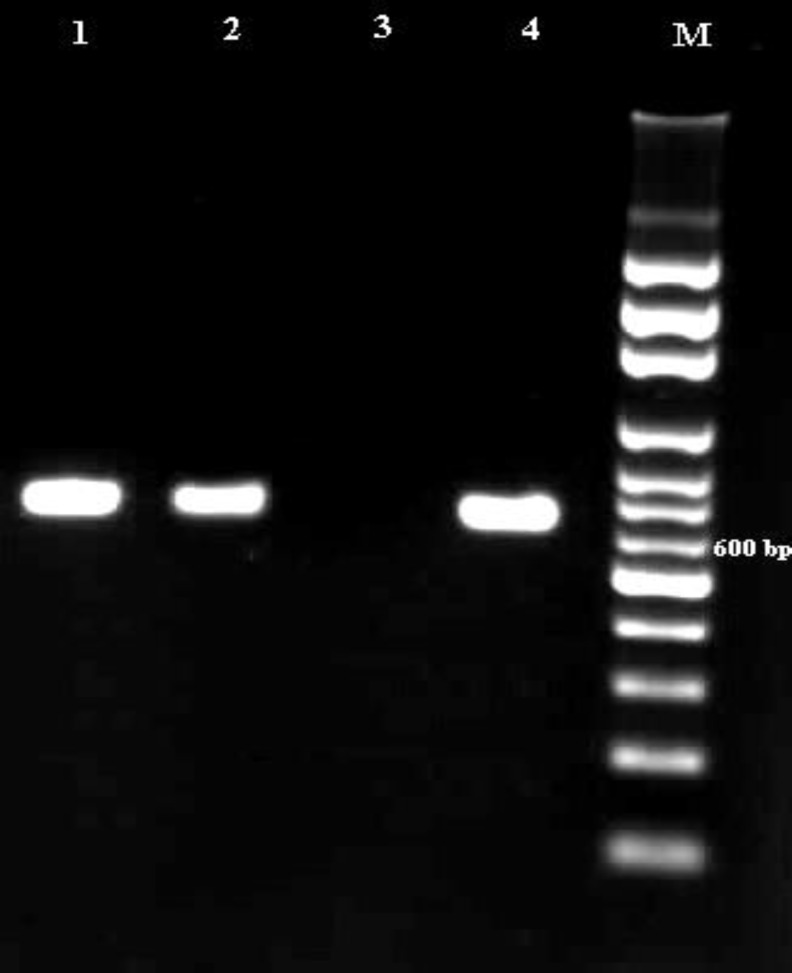
Agarose-gel electrophoresis of polymerase chain reaction (PCR) products amplified with genomic DNA from adult worms of *D. immitis* samples. **Lanes 1 and 2:** PCR products of *D. immitis* samples; **Lanes 3:** Negative control; **Lane 4:** Positive control (*D. immitis*); and **Lanes M:** 100-bp DNA marker

Adult worms of *D. immitis* were collected from heart of two necropsied dogs (Fig. 2). The two isolates successfully presented amplification of about 689 bp for the COX1 gene (Fig. 3). The sequences were achieved and compared to other available sequences in GenBank, using the BLAST system. The isolates had high similarity (more than 95.00%) with *D. immitis *GenBank references sequences. The COX1 sequences of the isolates obtained in this study were submitted to GenBank database (accession numbers: KT318126 and KT960976). The BLAST analysis indicated that the sequences of the isolates of *D. immitis* from this study were identical and presented 100% homology with* D. immitis* isolated from jackal (KT351852) in northeast Iran,^26^ dog (EU159111) and red panda (EU169124) in China, dog (KC107805) in Bangladesh and cat (AM749227), dog (AM749228) and wolf (DQ358815) in Italy and was 99.40% similar in that of *D. immitis* isolated from dog in Italy (AM749228; 3 bp differences in 561 bp).

All the taxa besides *Ascaris lumbricoides *(Accession no. AB591801) as out-group were included in a big branch tree. Phylogenetic analysis showed that *D. immitis* and *D. repens* were sister species.

**Fig. 4:**
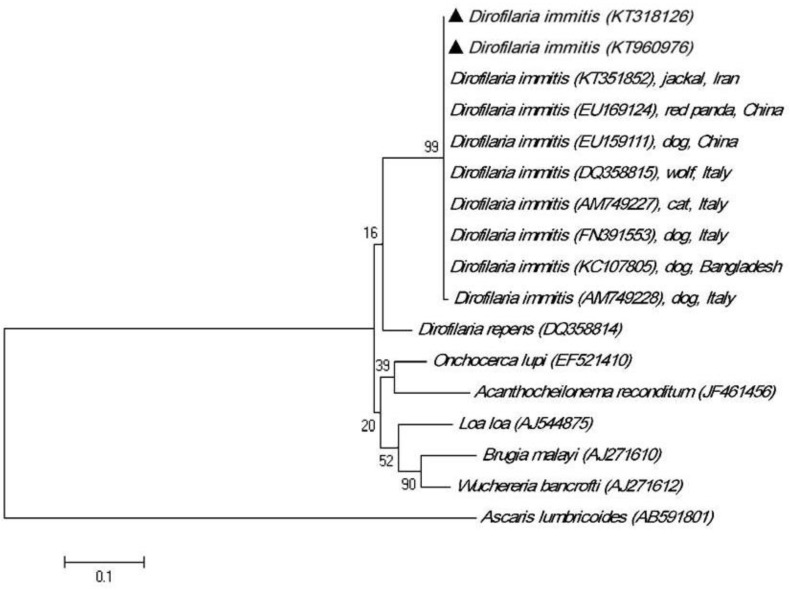
Phylogenetic tree of COX1 sequences of dog isolates of *Dirofilaria immitis* from Iran (▲) using maximum likelihood method based on the Tamura-Nei model in MEGA software. *Ascaris lumbricoides* (Accession no. AB591801) was as out group

Intra-species variation within isolates of *D. immitis* was 0 to 0.50%; meanwhile, inter-species sequence differences among the seven nematodes of onchocercidae family were significantly higher, being 10.70 to22.50% (Fig. 4).

## Discussion

Canine dirofilariasis has a cosmopolitan distribution. Meshkin-Shahr in northwest of Iran is presented as a high prevalent area for heartworm infection in dogs, in addition to documentation of human dirofilariasis cases.^16^^,^^18^^,^^19^

In the current study, based on the thin and thick blood smears examination, microfilariae of *D. immitis* were detected in 20.80% of the dogs. Different range of infection rates of canine dirofilariasis have been reported previously from Iran (1.4-51.42%), depending on the environmental and climatic conditions, distribution of the vector and diagnostic methods.^8^^-^^14^^,^^16^^,^^17^ The gold standard technique for canine dirofilariasis considered to be Knott’s test,^27^ thus, the real infection rate in the study area might be higher.

The results of this study showed that male dogs were more infected with *D. immitis* than that of females (16.40% versus 12.00%), however, similar to some previous studies,^13^^,^^16^^,^^17^^,^^28^ this difference was not statistically significant. This was in part probably due to sample size of the present study. Some other researchers, however, reported that rate of infection in males was higher than female dogs.^23^^,^^29^

Our findings indicated that the chance of infection with *D. immitis* was increased with age of the dogs and it was in agreement with some previous studies.^16^^,^^30^ Statistical analysis revealed greater infection rate in older dogs (more than two years old) than younger dogs (28.30% versus 6.40%) and it was possibly due to the longer exposure of older dogs to the mosquito bites.^31^

Sequence-based phylogenetic analysis is a useful tool to get information from organism evolutionary relationships. The existence of genetic variation among nematodes has been confirmed previously.^32^^-^^34^ However, only a few studies have analyzed molecular characterization of *D. immitis.*^35^^,^^36^ This study is the first phylogenetic analysis of *D. immitis* from Iran. As mentioned earlier, the isolates of *D. immitis* collected from dogs in Meshkin-Shahr had 100% homology with the isolates of *D. immitis* collected from dog, red panda, cat, jackal and wolf from different areas of the world except for one isolate of *D. immitis* from Italy (Accession no. AM749228) with 99.40% similarity.

Therefore, phylogenetic analysis reveals that variation of the host was not associated with sequence variation of the parasite isolates.

In the present study, the isolates of *D. immitis* were classified close to the cluster containing *D. repens* isolate. These results suggested the existence of polymorphic variation in the COX1 sequence in *Dirofilaria* species that could be suitable for species identification of the genus *Dirofilaria*. According to Huang *et al.* phylogeny analysis of the family Onchocercidae based on COX1 gene is in agreement with the classification of filarial nematodes using morphological and biological characters and could be helpful for distinguishing intra- and interspecies genetic variation.^35^

In conclusion, this study demonstrated that COX1 gene sequences were suitable markers for phylogenetic analysis of *Dirofilaria* nematodes. For an intensive understanding of genetic variation among populations of *D. immitis*, analysis of more isolates from other geographical areas, different hosts and genetic markers is recommended.
